# Preliminary Study on the Positive Expression Regulation of Alpha2-Macroglobulin in the Testicular Tissue of Male Mice by Environmental Estrogens

**DOI:** 10.3390/ijms252413434

**Published:** 2024-12-15

**Authors:** Hong-Mei Li, Yan-Rong Gao, Chang Liu, Yu-Xin Sheng, Ya-Jia Pu, Jia-He Sun, Ya-Nan Tian, Li Yang, Hui-Ming Ma, Hai-Ming Xu

**Affiliations:** 1 The Key Laboratory of Fertility Preservation and Maintenance of the Ministry of Education, Ningxia Medical University, Yinchuan 750004, China; lihongmei@nxmu.edu.cn (H.-M.L.); 20220310121@nxmu.edu.cn (Y.-R.G.); liuchang813909@163.com (C.L.); 230230020186@nxmu.edu.cn (Y.-X.S.); 240230020194@nxmu.edu.cn (Y.-J.P.); sjh919881051@outlook.com (J.-H.S.); tianyanan2434@163.com (Y.-N.T.); 2 School of Basic Medicine, Ningxia Medical University, Yinchuan 750004, China; 3 School of Public Health, Ningxia Medical University, Yinchuan 750004, China; 4 Laboratory Animal Centre, Ningxia Medical University, Yinchuan 750004, China; 5 The Key Laboratory of Environmental Factors and Chronic Disease Control, Ningxia Medical University, Yinchuan 750004, China

**Keywords:** environmental estrogens (EEs), alpha2-macroglobulin (A2M), estrogen receptor (ER), male reproductive system, interleukin-6 (IL-6)

## Abstract

The male reproductive impairment caused by environmental estrogens (EEs) stands as a pivotal research area in environmental toxicology. Alpha2-macroglobulin (A2M) emerges as a promising molecule capable of counteracting oxidative stress induced by EEs. This study conducted exposure experiments spanning PND1 to PND56 employing ICR mice, aiming to delve into the expression patterns of A2M and its modulated IL-6 in the testicular tissue of mice subsequent to diethylstilbestrol (DES) and benzophenone (BP) exposure, while elucidating the pivotal role of ERs in this intricate process. Our findings revealed that upon DES exposure (10 and 100 nM), there was a pronounced upregulation of A2M (mRNA and in situ protein levels) in mouse testicular tissue. Similarly, exposure to BPs (BP-1, BP-2, and BP-3, each at 10 and 1000 nM) exhibited comparable effects and increasing A2M levels in serum. Notably, BP exposure also caused an elevation in IL-6 levels (which could be directly regulated by A2M) within testicular tissue (mRNA and in situ protein). Remarkably, the specific estrogen receptor antagonist ICI 182780 (0.5 mg/kg/day) was effective in reversing the upregulation of both A2M and IL-6 induced by BP exposure. Significantly, the results of theoretical prediction of the potential ERE site in the A2m gene promoter region and ChIP-qPCR experiment provide essential and strong evidence for the key conclusion that A2m is the target gene of ER. Taken together, our study highlights EEs’ ability to regulate A2M expression in the male reproductive system via the ER signaling pathway. This vital insight deepens our understanding of molecular mechanisms protecting against oxidative stress caused by EEs.

## 1. Introduction

Environmental estrogens (EEs) are a class of exogenous chemicals that activate or inhibit the endocrine system after entering the body, thereby disrupting the stability of the internal environment and hormonal regulation. In recent years, EEs have garnered increasing attention as a key research area in environmental sciences. These chemicals, which mimic or disrupt natural estrogen, are increasingly found in various industrial goods, pesticides, plastics, and personal care products. EEs can enter the human body through diet, air, and skin contact, and are easily accumulated in living organisms. Long-term and extensive exposure to EEs can have adverse effects on the health and reproduction of humans, livestock, wildlife, etc. [1,2,3]. EEs disrupt the homeostasis of endogenous reproductive hormones mainly by binding to estrogen receptors (ERs), thereby interfering with the reproductive system and developmental processes of organisms [4,5]. The male reproductive damage effects caused by EEs are mainly manifested as incomplete testicular development, decreased sperm count and quality, and even the appearance of feminized gonadal phenotype [6,7].

Benzophenone-type UV filters (BPs) are widely used in personal care products and industrial applications to protect against UV radiation [1,8]. BP-1, BP-2, and BP-3, especially BP-3, are commonly found in various products, leading to their frequent presence in the environment matrix [9]. Taking environmental water bodies as an example, the highest recorded concentration of BP-3 in seawater is up to 1.395 mg/L in Hawaii, USA [10]. Considering the escalating detection of BPs in environmental water bodies with each passing year, the exposure via drinking water is a significant pathway that cannot be overlooked. This does not, however, preclude their potential application within the human body [11]. It is worth noting that BPs have been detected in humans in recent years, with increasing internal exposure concentrations, causing concerns about health risks [12,13]. A large number of computational models [14] and exposure experiments utilizing fish [15,16], amphibians [17], and rodents [18,19] as test subjects have consistently revealed estrogenic effects from BPs, with vitellogenin production and uterotrophic bioassays serving as prominent examples. Our earlier research indicated that BP exposure at environmentally relevant concentrations (10 and 1000 nmol/L) could suppress testicular differentiation and promote feminizing effects in the South African clawed toad (*Xenopus laevis*) [20]. Remarkably, a representative study conducted by Santamaria et al. showed that maternal mice exposed to 50 mg/kg b.w/day of BP-3 for 6 days exhibited an increased female sex ratio [21]. The latest research by our group shows that BPs can induce reproductive damage in male mice by regulating the ER/CCL27/ROS axis (using the classic environmental estrogen diethylstilbestrol, DES, as a positive control). Based on this research, the next question we are considering is which endogenous molecules can resist oxidative damage caused by these ubiquitous EEs [22].

Alpha2-macroglobulin (A2M), recognized for its ability to promote the proliferation of various cell types in vitro, also functions as a broad-spectrum protease inhibitor. It exhibits anti-inflammatory properties and a self-defense mechanism akin to that of leukocytes, potentially playing a crucial regulatory role in the inflammatory response in vivo. Consequently, it may indirectly contribute to the alleviation of oxidative stress [23]. A2M assists in the clearance of oxygen free radicals and combats oxidative stress to a significant degree, indicating that A2M may contribute to the protection of cells from oxidative damage [24]. Furthermore, A2M has the ability to bind to a range of cytokines and growth factors, thereby modulating their activity. It is a specialized secretory protein in the male reproductive system [25]. On one hand, A2M can regulate the inflammatory response and alleviate oxidative stress. On the other hand, it can enable the testes to perform normal spermatogenic and androgenic functions by reducing the concentration of TGF-β and prolonging the action time of IL-6 [26,27]. Collectively, A2M plays a pivotal role in stimulating cell growth, suppressing inflammation, and regulating oxidative stress. Drawing from existing research, we hypothesize that EEs can inflict oxidative harm on the male reproductive system, and A2M may partially alleviate this damage, serving a protective role in the body.

In this research endeavor, ICR mice served as our experimental subjects for exposure studies spanning from PND1 to PND56, which is a critical period for male reproductive development, in order to uncover the expression patterns of A2M and its regulatory factor IL-6 in the testicular tissue of mice following exposure to EEs, and to explore the potential contribution of ER in this process. Specifically, animal experiments are divided into two parts. In the first part, the experimental objective is to study the impact of BP-3 on male mice, focusing on A2M and related biomarkers, and in the second part, the research objective is to reveal whether BP-3 disrupts testicular development via the ER signaling pathway.

## 2. Results

### 2.1. Relative Expression Levels of A2m Gene in Mouse Ovaries and Testes

The relative expression level of the *A2m* gene in mouse ovaries was higher than that in testes (Figure S1A, data retrieved from BioGPS database). The results of a RT-qPCR experiment further confirmed this (Figure S1B). These results suggest that *A2m* may be associated with signaling pathways regulated by estrogen and estrogen receptors.

### 2.2. Exposure to DES Leads to Upregulation of A2M Expression Testes of Mice (PND1–PND56)

**Figure 1:**
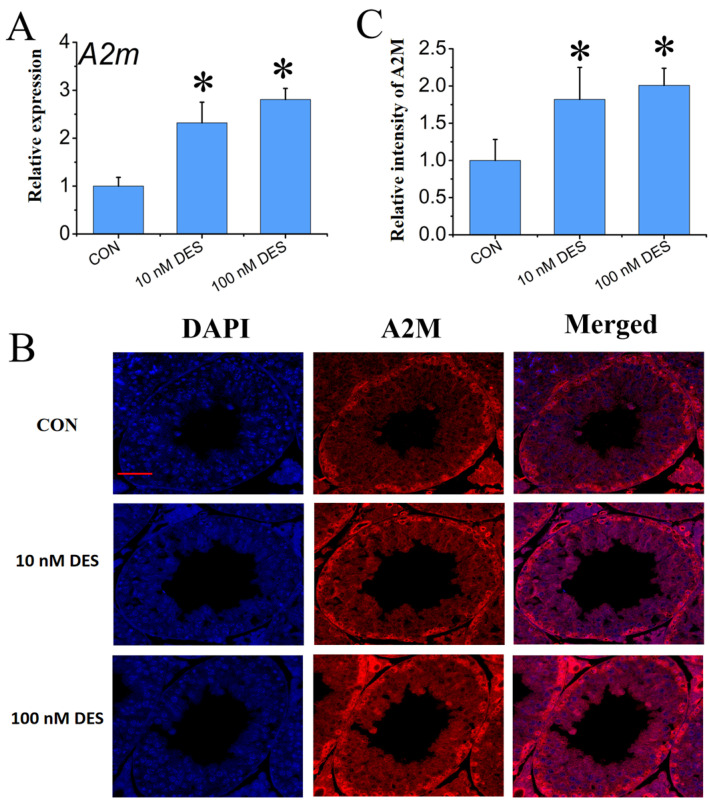
The effect of DES exposure on the expression level of A2M in mouse testes (PND1–PND56) (**A**). Relative mRNA expression level (**B**). In situ protein expression level (IF assay) (**C**). Quantitative analysis of IF assay results. Scale bar: 50 μm. Data are expressed as mean ± SD. * Indicates significant differences between BP groups and the control group (*p* < 0.05).

The mRNA levels of *A2m* were significantly elevated in the presence of 10 and 100 nM DES, as compared with the testes of mice at PND 56 in the control group (*p* < 0.05) (Figure 1A). Figure S2 shows the immunofluorescence (IF) negative control result, indicating good experimental quality control. At the protein level in situ, multiple IF assays utilizing specific cell markers for Sertoli cells (SOX9) and spermatocytes (DAZL) revealed that A2M is localized in the cytoplasm of germ cells, Leydig cells, and Sertoli cells within the seminiferous tubules, with a marked increase in A2M within the DES-treated groups, indicating respond to estrogen exposure (Figure 1B and Figure S3). Upon further analysis, the relative intensity of A2M was found to be significantly upregulated by 10 and 100 nM DES in the testes of mice at PND 56 relative to the control (*p* < 0.05) (Figure 1C).

### 2.3. Exposure to BPs Increased A2M Level in the Testes of Mice (PND1–PND56)

**Figure 2:**
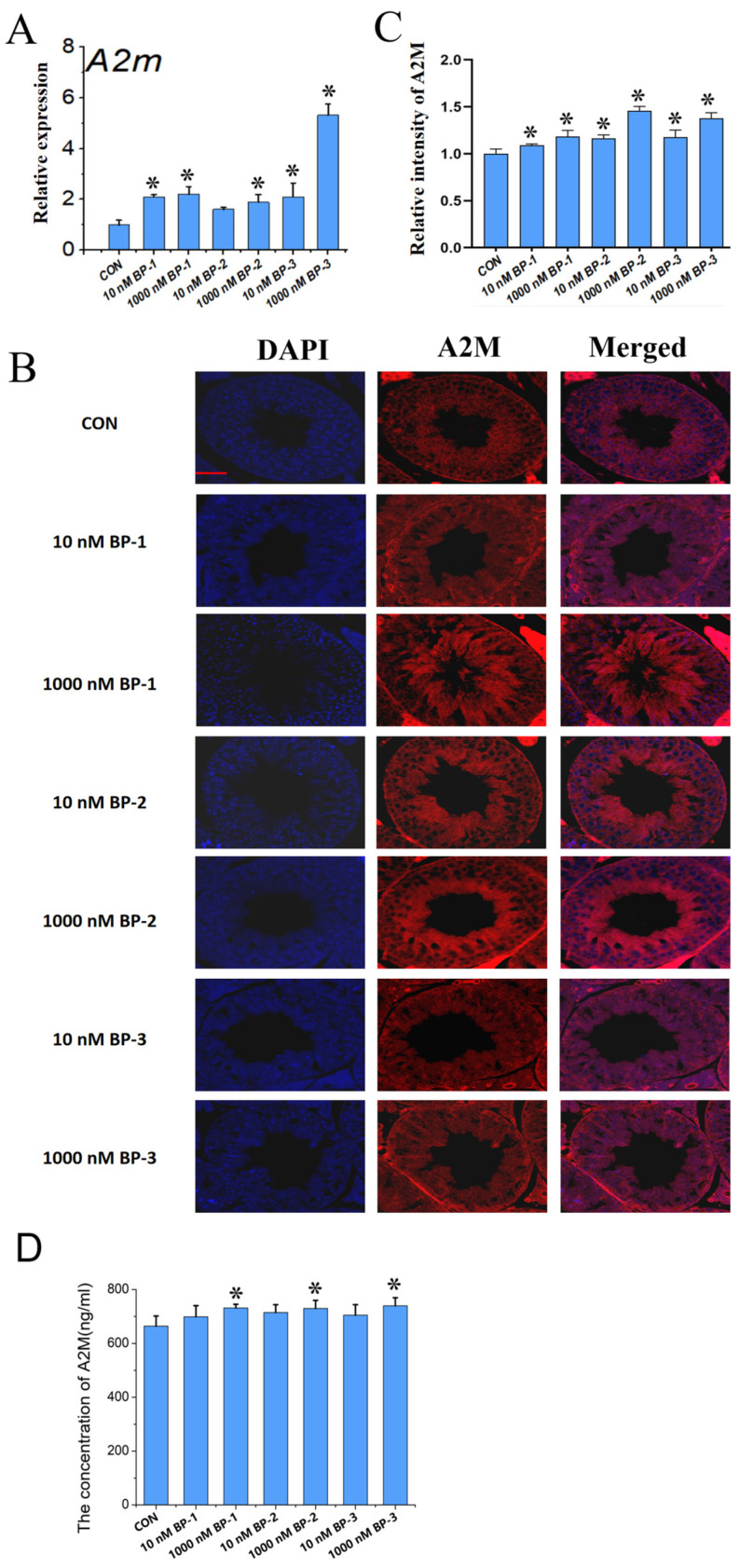
The effect of BP exposure on the expression level of A2M in mouse testes (PND1–PND56) (**A**). Relative mRNA expression level (qPCR). (**B**). In situ protein expression level (IF assay). (**C**). Quantitative analysis of IF assay results. (**D**). The concentration of A2M in serum. Scale bar: 50 μm. Data are expressed as mean ± SD. * Indicates significant differences between BP groups and the control group (*p* < 0.05).

The mRNA levels of *A2m* in all doses of BP-1, BP-2, and BP-3 were significantly elevated compared with those in the control mouse testes at PND 56 (all *p* < 0.05), with the exception of 10 nM BP-2 (Figure 2A). At the in situ protein level, the fluorescent signal of A2M expression showed an increasing trend in all doses of BP-1, BP-2, and BP-3 relative to the control (Figure 2B). The quantitative analysis results indicate that the relative intensity of A2M was significantly increased in a dose-dependent manner in the mouse testes compared with the control (*p* < 0.05) (Figure 2C). In addition, the measured concentrations of A2M were higher in serum samples of mice at PND 56 following exposure to all doses of BP-1, BP-2, and BP-3 than those in the control, with significant difference in the 1000 nM BP-1, BP-2, and BP-3 groups (*p* < 0.05) (Figure 2D).

### 2.4. Exposure to BPs Resulted in Upregulation of IL-6 in the Testes of Mice (PND1–PND56)

**Figure 3:**
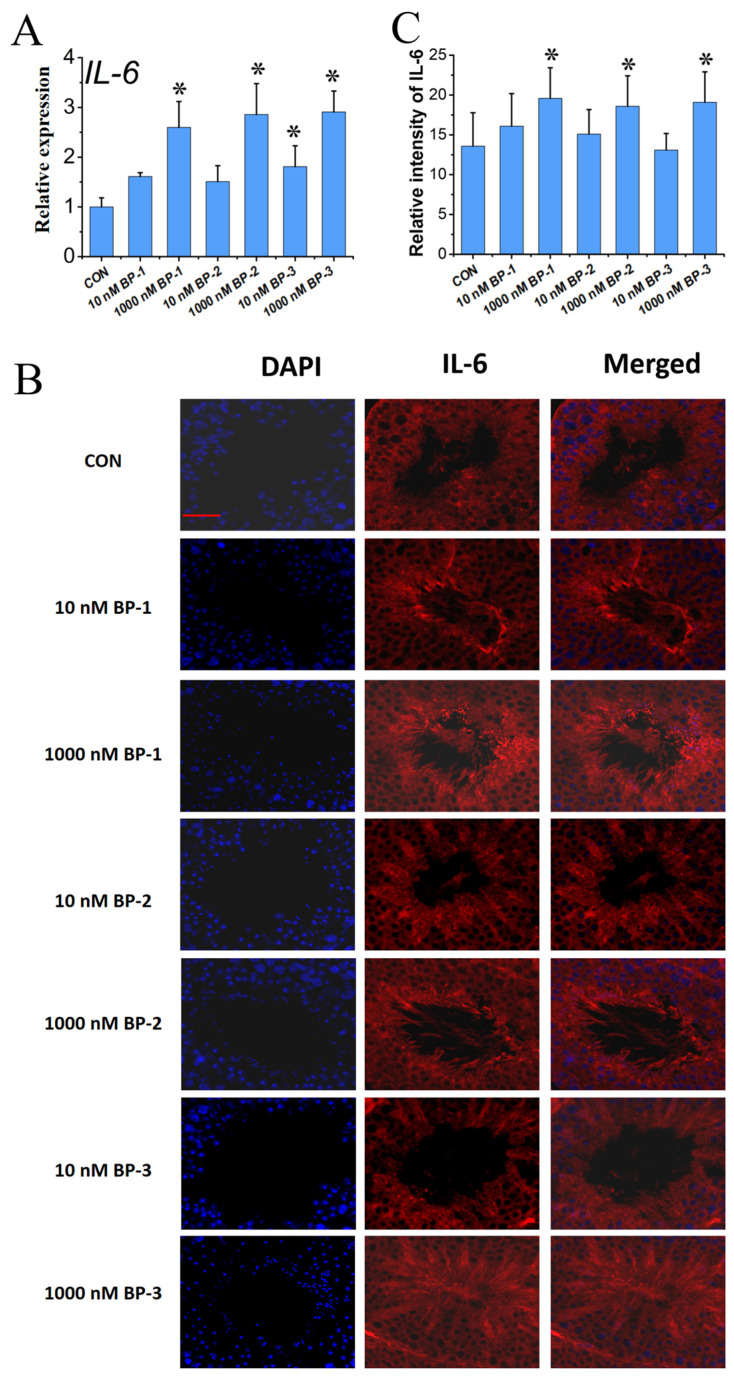
The effects of BP exposure on the expression level of IL-6 in mouse testes (PND1–PND56) (**A**). Relative mRNA expression level (qPCR). (**B**). In situ protein expression level (IF assay). (**C**). Quantitative analysis of IF assay results. Scale bar: 50 μm. Data are expressed as mean ± SD. * Indicates significant differences between BP groups and the control group (*p* < 0.05).

The mRNA expression of IL-6 was found to be significantly elevated in the testes of mice treated with all dosages of BPs compared with the control group at PND 56 (*p* < 0.05) (Figure 3A). The in situ protein expression of IL-6 was found to be superimposable with A2M and was localized to the cytoplasm of germ cells, Leydig cells, and Sertoli cells within the testis, as demonstrated by double IF experiments. Furthermore, the levels of IL-6 exhibited an increasing trend following exposure to 10 and 1000 nM concentrations of BPs compared to the control group, a finding that is supported by quantitative analysis (*p* < 0.05) (Figure 3B,C and Figure S3B).

### 2.5. ICI 182780 Intervention Could Counteract the Upregulation of A2M and IL-6 Expression in the Testes of Mice Caused by BP Exposure (PND1–PND56)

**Figure 4:**
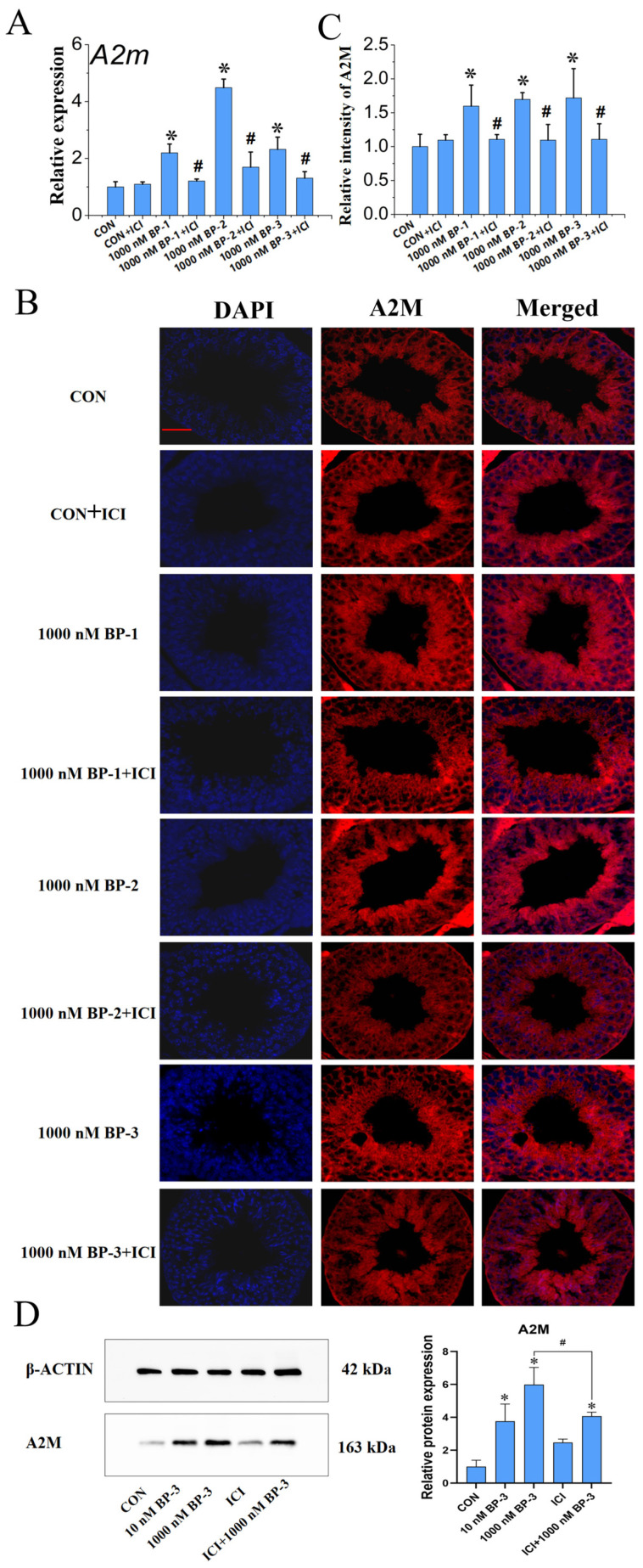

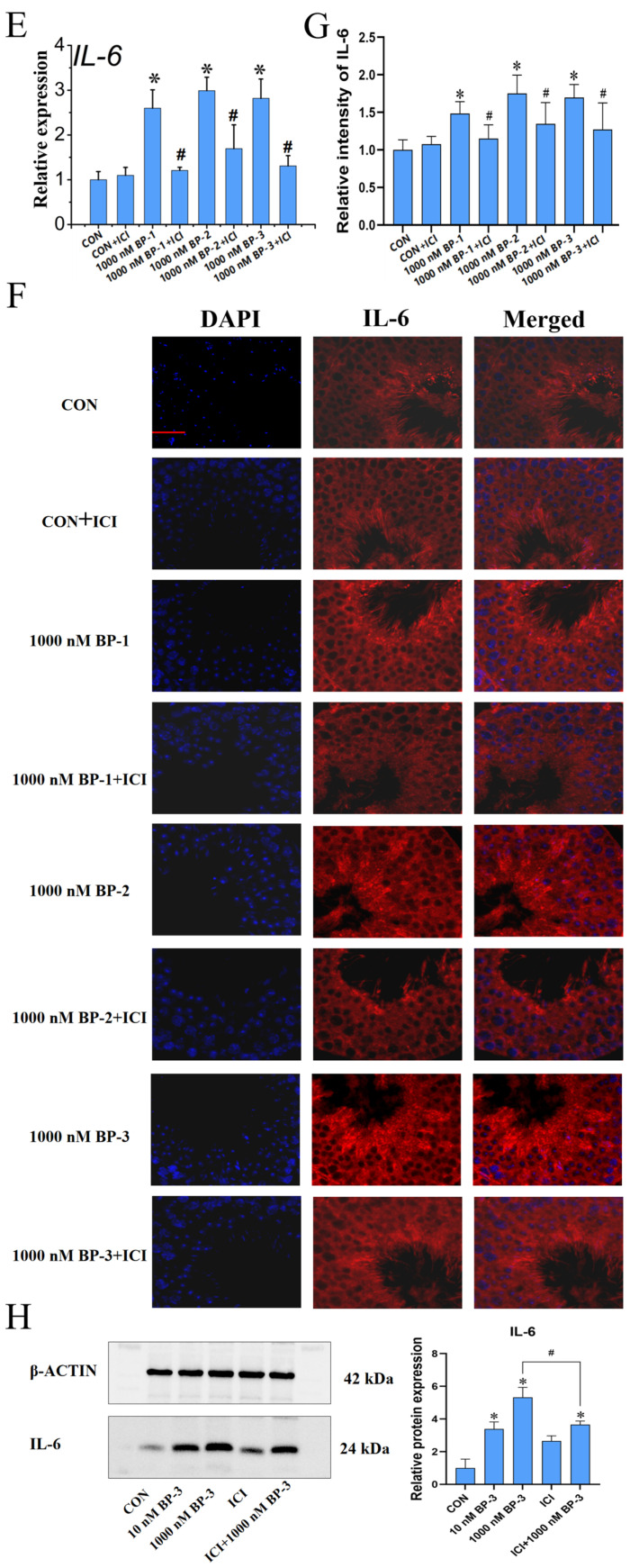
The effects of ICI intervention on the upregulation of A2M and IL-6 in testicular tissue of mice exposed to BPs. (**A**–**C**) Relative mRNA expression level (qPCR), in situ protein expression level (IF assay), and the quantitative analysis of IF assay results of A2M, respectively. (**D**–**F**) Relative mRNA expression level (qPCR), in situ protein expression level (IF assay), and the quantitative analysis of IF assay results of IL-6, respectively. (**G**,**H**) Protein quantitative expression levels (WB assay), and the quantitative analysis of WB assay results of A2M and IL-6, respectively. Scale bar: 50 μm. Data are expressed as mean ± SD. * Indicates significant differences between BP groups and the control group (*p* < 0.05). # Indicates significant difference between BPs groups and the corresponding ICI 182780 intervention groups (*p* < 0.05).

Figure 4A–D showed the antagonistic effect of ICI 182780 intervention on the upregulation of A2M in mouse testicular tissue induced by exposure to BPs. Compared with the control, A2M expression in male mouse testes at PND 56 was significantly increased by exposure to BP-1, BP-2, and BP-3 at 1000 nM (*p* < 0.05). The expression of A2M was not significantly affected by ICI 182780 exposure. It is worth noting that ICI 182780 intervention could significantly antagonize BP-induced upregulation of A2M mRNA expression (*p* < 0.05) (Figure 4A). Similarly, at in situ protein levels, it appeared that the increased A2M expression induced by 1000 nM BP-1, BP-2, and BP-3 could be antagonized by ICI 182780 (Figure 4B), which was further confirmed by the quantitative analysis of fluorescence signals (Figure 4C). Using WB, we found that 10 and 1000 nM BP-3 upregulated A2M protein, with 1000 nM showing a significant increase compared with the controls. ICI 182780 alone had no effect, but it significantly reversed BP-3’s effect at 1000 nM (*p* < 0.05) (Figure 4D).

Figure 4E–H represent the antagonistic effect of ICI 182780 intervention on the upregulation of IL-6 in mouse testicular tissue induced by BP exposure. Exposure to 1000 nM concentrations of BPs resulted in a significant increase in the relative expression of IL-6 compared with the control group in testes of mice at PND 56 (*p* < 0.05). The expression of IL-6 was not significantly altered by exposure to ICI 182780 alone. Significantly, ICI 182780 intervention could significantly reverse the upregulation of IL-6 gene expression caused by 1000 nM BPs (*p* < 0.05) (Figure 4E). Similarly, at the in situ protein level, the presence of ICI 182780 appeared to counteract the increased effects of 1000 nM BPs on IL-6 expression (*p* < 0.05) (Figure 4F). Further analysis revealed that ICI 182780 significantly reduced the relative intensities of IL-6 in response to BP exposure (*p* < 0.05) (Figure 4G). WB analysis revealed that 10 and 1000 nM BP-3 increased A2M protein levels, with 1000 nM BP-3 showing significant changes. ICI 182780 alone had no effect, but it significantly reversed BP-3’s effect at 1000 nM (*p* < 0.05) (Figure 4H).

### 2.6. Functional ERE Site Was Indeed Located in the A2m Gene Promoter Region of Mice

**Figure 5:**
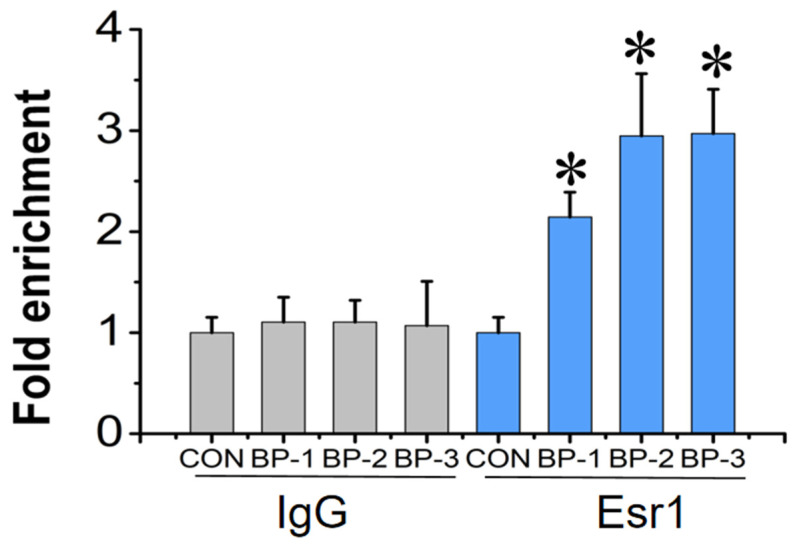
Quantitative enrichment of *A2m* after chromatin immunoprecipitation (ChIP) with Esr1antibody and immunoglobulin G (IgG) in mouse testes following postnatal exposure to 1000 nM BPs (BP-1, BP-2, and BP-3) for 56 days. Data were expressed as mean ± SD. * Indicates significant difference between BP groups and the control group (*p* < 0.05).

Utilizing the UCSC Genome Browser Home database, we speculated that there were theoretically several ERE sites in the *A2m* gene promoter of mice (Figure S4). Based on this, the ChIP-qPCR primer was designed by selecting the ERE site with the highest probability (minimum *p*-value) (Figure S5). ChIP-qPCR analysis revealed that in the group treated with 1000 nM BPs, the primer targeting the binding region site (from −397 to −382) significantly amplified ESR1-bound DNA fragments compared with the control group (*p* < 0.05) (Figure 5).

## 3. Discussion

Numerous studies have shown that EEs can cause male reproductive damage through various pathways, with oxidative damage being one of the main routes [28,29,30,31,32]. Therefore, it is crucial to search for molecules (a classic example is Nrf2) that have potential protective effects against cellular oxidative damage [33]. In the preliminary research conducted by our research group, candidate molecule *A2m* was successfully obtained based on transcriptome sequencing. It is widely known that A2M is a broad-spectrum protease inhibitor that plays an important regulatory role in the inflammatory response in the body and can effectively alleviate oxidative stress [24]. Based on this, here, we focused on investigating the effects of classical DES and novel BP exposure on this molecule, as well as the potential role of estrogen receptors in it.

A2M is an important protein that can induce and regulate immune responses, participate in cellular signal transduction, and regulate protease activities, playing a crucial role in various human physiological activities [23,34,35,36]. Here, we focus on the relationship between A2M and the reproductive system. A2M is closely related to sperm quality, oocyte growth and maturation, ovulation, and embryo implantation [25]. The relationship between A2M and sperm quality is mainly reflected as follows: A2M can facilitate normal spermatogenic and androgenic functions in the testes by reducing the concentration of TGF-beta and prolonging the action time of IL-6 [27,28]. Specifically regarding the regulation of IL-6 by A2M, on one hand, A2M can inhibit the cleavage of diverse endopeptidases and protect IL-6 from degradation, and on the other hand, A2M can bind to IL-6 but does not inhibit the binding of IL-6 to its receptor. In this study, exposure to BPs upregulated IL-6 levels in mouse testicular tissue, suggesting that A2M may play a bridging molecule (mediator) role.

ER signaling pathway is the primary route for EEs to disrupt endocrine function [37,38]. In this study, we found that the addition of ICI 182780 could largely counteract the upregulation of A2M and IL-6 expression caused by BPs, suggesting that ER mediates the aforementioned effects. In the initial research design, we attempted to perform molecular docking between the studied compounds and A2M. The first round of docking results showed that these compounds and A2M could not form stable interactions, indicating structural mismatch or a lack of suitable binding sites. The second round of docking results after adjusting the molecular structure showed that stable interactions could still not be formed, indicating that these compounds do not have suitable binding sites with A2M. Therefore, the possibility of direct interaction between these compounds and A2M could theoretically be ruled out. It is noteworthy that the results of theoretical prediction of the potential ERE site in the A2m gene promoter region and the ChIP-qPCR experiment provide essential and strong evidence for the key conclusion that *A2m* is the target gene of ER. Similarly, following exposure to estradiol (E2) in *X. laevis*, *A2m* was identified as a feminized estrogen receptor (ER) target gene [39].

Given the breadth and importance of A2M function, it can be reasonably inferred that this molecule mediates the regulation of many key signaling pathways. However, there is limited literature on this topic. As one of the most commonly used bioinformatics databases internationally, KEGG provides powerful support for deep analysis of omics data with its precise biological pathway mapping and rich annotations. Therefore, a search was conducted using the KEGG database as the target database and *A2m* as the keyword. The results showed that this molecule is involved in the map04610 pathway (complement and coagulation cascades), which provides insights for future research.

The shortcomings of this study are mainly reflected in the following two points. Firstly, there was no further validation of the research results using estrogen receptor gene knockout mice or low-expression cell models. Secondly, it has only been preliminarily identified that *A2m* is the target gene of ER, without in-depth exploration and verification of the downstream regulatory network of *A2m*. Next, animal models or cell models of estrogen receptor gene knockout can be used to further confirm the experimental results of this study. The methods of omics sequencing, bioinformatics analysis, and network data mining can be used to construct the upstream and downstream molecular regulatory network of *A2m* in the male reproductive system. More in-depth investigations (including but not limited to environmental epidemiology and toxicology) can be conducted to explore the possibility of using A2M as a biomarker for the effects of EE exposure. Furthermore, exploring this indicator as an effective intervention target in order to alleviate male reproductive damage caused by environmental factors through preventive interventions is of great significance.

## 4. Materials and Methods

### 4.1. Animal Rearing

SPF ICR mice were procured from the Experimental Animal Facility of Ningxia Medical University. Each pregnant mouse was individually placed in an IVC cage and kept under a rigorously controlled environmental temperature (23 ± 1 °C), relative humidity (40–50%), and regulated 12 h light–dark cycle. The mice had unrestricted access to food and water throughout the experiment. This research protocol has undergone a thorough review process and has received formal approval (AEWC-NXMU-2023-G038).

### 4.2. Chemical Exposure Determination

For in vivo experiments, we systematically reviewed and analyzed the reported concentrations of DES and BPs in diverse environmental water bodies from the global literature. These concentrations range from a few nanograms to several milligrams per liter, serving as a crucial benchmark for establishing the exposure dose of 10 and 1000 nM BP-1 (2.14–214 μg/L, 98% purity), BP-2 (2.46–246 μg/L, 98% purity), and BP-3 (2.28–228 μg/L, 98% purity) from Amethyst Chemical (Beijing, China) with 10 and 100 nM DES (2.68–26.8 μg/L, 99% purity) from Dr. Ehrenstorfer GmbH, Augsburg, Germany, used in vivo experiments conducted in this research.

### 4.3. Experiment Design

Experiment I aimed to study the impact of BP-3 on male mice, focusing on A2M and related biomarkers. Strictly following the randomization principle, the experimental animals were divided into the solvent control group (DMSO, 0.01% *v*/*v*), DES groups (10 and 100 nM), and BP groups (BP-1, BP-2, BP-3; 10 and 1000 nM). A group of 6 dams, each with their male offspring on postnatal day 1 (PND 1), were used for exposure experiments. Before and after weaning, the offspring mice were exposed to the above compounds through the routes of breast milk and drinking water, respectively. The exposure experiment continued until PND56. After exposure, the mice were humanely euthanized for specimen collection.

Experiment II investigated whether BP-3 disrupts testicular development via the ER signaling pathway. First, neonatal mice were exposed to BP-3 (the exposure routes were the same as Experiment I) and/or ER antagonist—ICI 182780 (intraperitoneally, 0.5 mg/kg/day) from PND 1 to PND 56. After exposure, the mice were anesthetized and sampled. Then, the mouse *A2m* gene promoter sequence was obtained using the UCSC Genome Browser. Subsequently, the theoretical ERE sites were predicted with the hTFtarget database. Last, ChIP-qPCR was used to verify whether the theoretical ERE site was functional.

The main reagents and kits used in this study are shown in Table S1.

### 4.4. RNA Extraction and RT-qPCR

Total RNA extraction, reverse transcription, and gene amplification were conducted following the manufacturer’s protocols. RT-qPCR was used to assess mRNA expression levels, with Gapdh as the reference gene. For more details on the methods and primers, refer to the Supplementary Materials (Table S2).

### 4.5. Immunofluorescence (IF) Staining

For IF staining, testicular tissue sections were placed on identical slides for comparison. Each group included at least 6 testes for analysis. Sections were incubated in 0.3% Triton X-100 in PBS for 15 min, blocked with 5% normal goat serum in PBS for 1 h at room temperature, and then incubated overnight at 4 °C with primary antibodies against A2M (ABclonal Technology, Wuhan, China, A9752, 1:200) and IL-6 (Wanleibio, WL02841, 1:200). After washing, sections were incubated with Cy3-conjugated secondary antibody for 1 h at room temperature in the dark, and mounted with DAPI-containing medium. Fluorescent images were acquired using a Leica DM2500 microscope, and fluorescent intensity was quantified using ImageJ. Slides were examined with a Nikon A1R SI confocal microscope, with two negative controls conducted.

### 4.6. Determination of the Concentration of A2M

The concentration of the total A2M in serum of mice at PND56 was determined by the commercial enzyme-linked immunosorbent assay kit from BIOMAC (Nanjing BYabscience technology, Nanjing, China) according to the method of Birkenmeser and Sugbrandd (1993).

### 4.7. Prediction of ERE Sequence in Mouse A2m Gene Promoter Region

The UCSC Genome Browser Home database was used to obtain the 2000 base sequence upstream of the start codon ATG of the mouse *A2m* gene (i.e., the promoter sequence of the *A2m* gene). The A base of the ATG start codon was defined as +1, and the nearest base before +1 was −1. Then, the sequence was saved in FASTA format. The above sequence was imported into the hTFtarget database, and ESR1 was entered into the “transcription factor” input box to predict the theoretical binding site between ESR1 and the mouse *A2m* gene. The theoretical ERE site with the highest ranking (the lowest *p*-value) was selected, and the ChIP-qPCR primer was designed using the NCBI Primer design tool (https://www.ncbi.nlm.nih.gov/tools/primer-blast/, accessed on 14 March 2024). SnapGene was used for plotting.

### 4.8. ChIP-qPCR

To investigate the potential for ER to directly modulate *A2m*, ChIP-qPCR analysis on the mouse testes was conducted. Following the protocol provided by Cell Signaling Technology, the testes were extracted, chopped, rinsed, crosslinked with formaldehyde, quenched with glycine, disrupted, lysed, nuclei isolated, DNA digested, and sheared to 150–250 bp fragments. A 2% aliquot served as the input control, and the rest was immunoprecipitated with anti-ERα antibody or IgG overnight at 4 °C. The next day, complex washing, chromatin elution, reverse crosslink with proteinase K, and DNA purification and resuspension proceeded sequentially. ChIP-DNA and input control were assessed by qPCR using SYBR Green Master Mix on a CFX96 Real-Time PCR System. The details of the experimental operations and primer information can be found in the Supplementary Materials.

### 4.9. Westem Blot Assay (WB)

Three milligrams of testicular tissue from each mouse group were sampled for total protein extraction using a whole protein extraction kit (KGB5303-100). Protein content was assessed with a BCA assay kit (KGB2101-1000), and proteins were denatured by boiling. Following SDS-PAGE and membrane transfer, the PVDF membrane was incubated with A2M (1:1500), IL-6 (1:1000), and β-actin (1:5000) antibodies at 4 °C overnight with shaking. After TBST washing, the membrane was incubated with species-specific secondary antibodies (1:20,000) for 1 h at room temperature. Membranes were washed again with TBST and developed with ECL, and target band intensities were quantified using Image J software to calculate relative protein expression levels.

### 4.10. Statistical Analysis

Statistical analysis was conducted utilizing SPSS 26.0 and graphically depicted via Origin Pro 8.5. Normally distributed data are presented in the form of mean ± standard deviation (SD). Group differences were assessed by means of one-way ANOVA with multiple comparisons, with statistical significance set at *p* < 0.05.

## 5. Conclusions

EEs (DES and BPs) can upregulate the expression level of A2M in the male reproductive system through the ER signaling pathway, and A2M can further increase the expression of IL-6, which plays a double-edged sword effect in the normal functioning of male reproductive function. These results suggest that the important role played by A2M should be taken seriously. As an endogenous molecule recognized nearly half a century ago, its role is not just that of a member of the traditional protease inhibitor family. Its potential physiological functions, including but not limited to regulating male reproductive function, should be re-evaluated. In addition, the pathway in which A2M participates and downstream molecules regulated by this molecule should also be continuously explored. Significantly, the results of this study can provide a new scientific perspective for a deeper understanding of the molecular mechanism of the body’s resistance to oxidative damage caused by harmful environmental factors.

## Data Availability

The original contributions presented in the study are included in the article/Supplementary Materials; further inquiries can be directed to the corresponding author/s.

## Supplementary Materials

The following supporting information can be downloaded at: https://www.mdpi.com/article/10.3390/ijms252413434/s1.
