# Supplementary material for: Preliminary Study on the Positive Expression Regulation of Alpha2-Macroglobulin in the Testicular Tissue of Male Mice by Environmental Estrogens
Source: Int J Mol Sci. 2024 Dec 15;25(24):13434. doi: 10.3390/ijms252413434 (PMC11676208; doi:10.3390/ijms252413434)
Supplement: Supplementary file 1 [file ijms-25-13434-s001.zip › ijms-3295092-supplementary.pdf]

Supplementary Materials

Preliminary Study on the Positive Expression Regulation of Alpha2-macroglobulin in the Testicular Tissue of Male Mice by Environmental Estrogens

Hong-Mei Li[1,2,3#], Yan-Rong Gao[1,2,3#], Chang Liu[1,2], Yu-Xin Sheng[1,2,3], Ya-Jia Pu[1,2,3], Jia-He Sun[1,2], Ya-Nan Tian[1,2], Li Yang[5], Hui-Ming Ma[1,2*], Hai-Ming Xu[1,3,4*]

[1] The Key Laboratory of Fertility Preservation and Maintenance of the Ministry of Education, Ningxia Medical University, Yinchuan, 750004, Ningxia, China

[2] School of Basic Medicine, Ningxia Medical University, Yinchuan, 750004, Ningxia, China

[3] School of Public Health, Ningxia Medical University, Yinchuan, 750004, Ningxia, China

[4] The Key Laboratory of Environmental Factors and Chronic Disease Control, Ningxia Medical University, Yinchuan, 750004, Ningxia, China

[5] Laboratory Animal Centre, Ningxia Medical University, Yinchuan, 750004, Ningxia, China

* Correspondence:

Hai-Ming Xu E-mail: xuhaiming1986@nxmu.edu.cn;

Hui-Ming Ma E-mail:

Materials and Methods

Table S1. The main reagents and kits used in this study.

**Reagents**	**Manufacturer**	**Catalog No.**
RNAsimple Total RNA Kit	TIANGEN Biotech (Beijing), China	DP419
FastKing gDNA Dispelling RT SuperMix Kit		KR118
RealUniversal Color PreMix (SYBR Green) Kit		FP205
Mouse α2-macroglobulin ELISA kit	Nanjing BYabscience Technology, China	BY-EM220986
SimpleChIP® Plus Enzymatic Chromatin IP Kit (Magnetic Beads)	Cell Signaling Technology, USA	#9005
IF-primary antibody-A2M	ABclonal Technology	A9752
IF-primary antibody-IL-6	Wanleibio	WL02841
IF-secondary antibody-Cy3 goat anti-rabbit IgG (H+L)	ABclonal Technology	AS007
CHIP-qPCR-antibody-ESR1	Invitrogen	MA127107
**Whole protein extraction kit**	Jiangsu Kaiji Biotechnology Co. LTD	**KGB5303-100**
**BCA assay kit**	Jiangsu Kaiji Biotechnology Co. LTD	**KGB2101-1000**
**ECL hypersensitive chemical developer**	Jiangsu Kaiji Biotechnology Co. LTD	**KGC4602-200**

RNA Extraction and RT-qPCR

RNA was extracted from the mouse testicular tissue (PND56) using the RNA prep Pure Reagent, adhering strictly to the manufacturer's instructions (Tiangen, Beijing, China). The quantity of RNA obtained was measured with a NanoDrop 2000 spectrophotometer (Thermo Fisher Scientific, USA). To assess the integrity of the RNA, agarose gel electrophoresis with a 1% gel stained with G-red nucleic acid dye (BioTeke Biotechnology, Beijing, China) was performed. Following this, 1 μg of total RNA was converted to complementary DNA (cDNA) utilizing the Fast Quant RT Kit's protocol. The gene expression was quantified with SYBR Green I on a CFX96 Real-Time PCR Detection System (Bio-Rad, USA). The primer sequences employed are outlined in Table S2. Before quantitative PCR (qPCR), and the primers' efficiency was confirmed to fall within the acceptable range of 90% to 110%. To ensure accuracy, the gene

expression levels were normalized against the housekeeping gene *Gapdh*. The relative gene expression changes were calculated using the $2^{-\Delta\Delta Ct}$ method.

Table S2. Primer sequences and relevant information used for RT-qPCR.

Gene Symbol	Accession No.	Primer Sequences (5′ - 3′)	Product Length
A2m	NM_001384258.1	F: AGATGGTGAGATTTCGTGTTGTC<br>R: ACGGTCCTGCCTGATTCTGTA	119
Gapdh	NM_008084.3	F: GCCTCGTCCCGTAGACAAAA<br>R: CAATCTCCACTTTGCCACTGC	104
IL-6	NM_008084.3	F: CCTCGTCCCGTAGACAAAA<br>R: CTGTTGTTCAGACTCTCTCCCT	

ChIP-qPCR

To ascertain A2M as a gene specifically targeted by ER, a ChIP-qPCR analysis on mouse testes was performed. Adhering meticulously to the protocol furnished by Cell Signaling Technology (Cat. No. #9005), the ChIP procedure was executed. The testes were meticulously extracted, minced in chilled PBS, and subsequently rinsed thoroughly with cooled PBS. Subsequently, a swift crosslinking reaction was induced with 1.5% formaldehyde in the presence of protease inhibitors, and the reaction was promptly quenched with glycine.

The testicular tissue was then mechanically disrupted using an MM301 grinder (RETSCH, Germany), and was lysed in a buffer enriched with dithiothreitol and protease inhibitors. Nuclei were isolated, resuspended, and subjected to micrococcal nuclease treatment to fragment DNA to an approximate size range of 150–300 base pairs (bp), halted by the addition of EDTA. The nuclei were further sonicated to shear the DNA into fragments ranging from 200 to 300 bp. A 2% aliquot of the sheared chromatin served as the input control, while the remaining chromatin underwent immunoprecipitation overnight at 4 °C with an anti-ERα antibody or IgG as a negative control. After incubation with protein G magnetic beads, the complexes were rigorously washed, and chromatin was eluted. Cross-links were reversed using proteinase K at 65 °C for 2 hours. The DNA was subsequently purified and resuspended in distilled water. The ChIP-enriched DNA and the 2% input control were quantitatively analyzed by qPCR using SYBR Green Master Mix (TIANGEN, Beijing, China) on a CFX96 Real-Time PCR Detection System. Specific primers designed to

amplify an 80–200 bp region surrounding the putative estrogen response element (ERE) of ER target genes of interest were employed.

Results

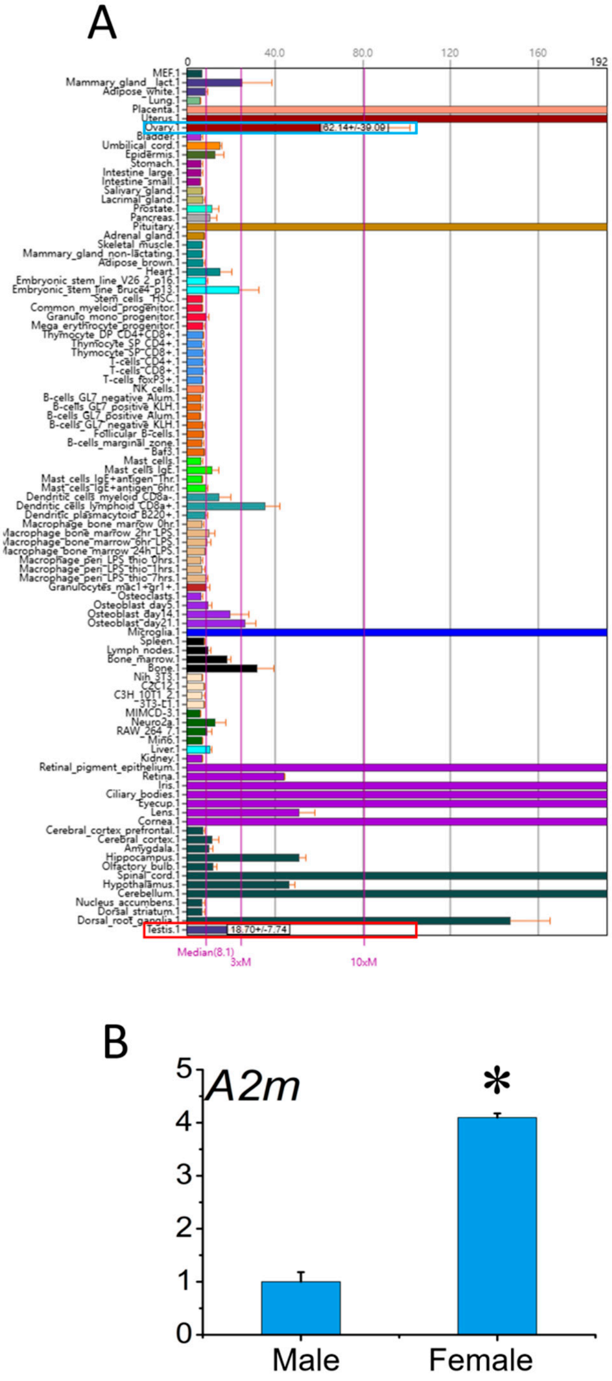

Figure S1. Data retrieved from the BioGPS database (A) and the relative expression (B) of A2M in the ovaries and testes of mice at PND 56. * Represents a significant difference between the males and females ($p < 0.05$).

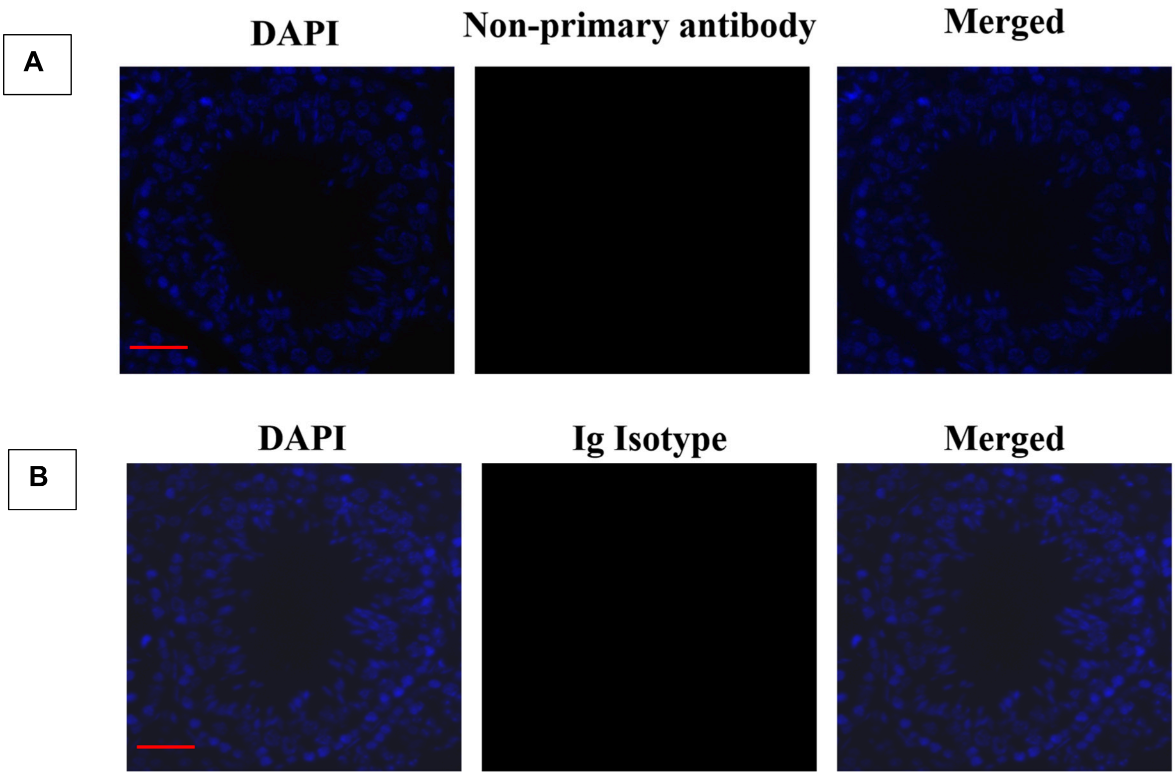

Figure S2. IF results of negative controls for each primary antibody in the testes of mice from the control group at PND56. (A) Obtained by omitting the primary antibody. (B) Obtained by using the non-immune Ig isotype. Scale bar: 50 μm.

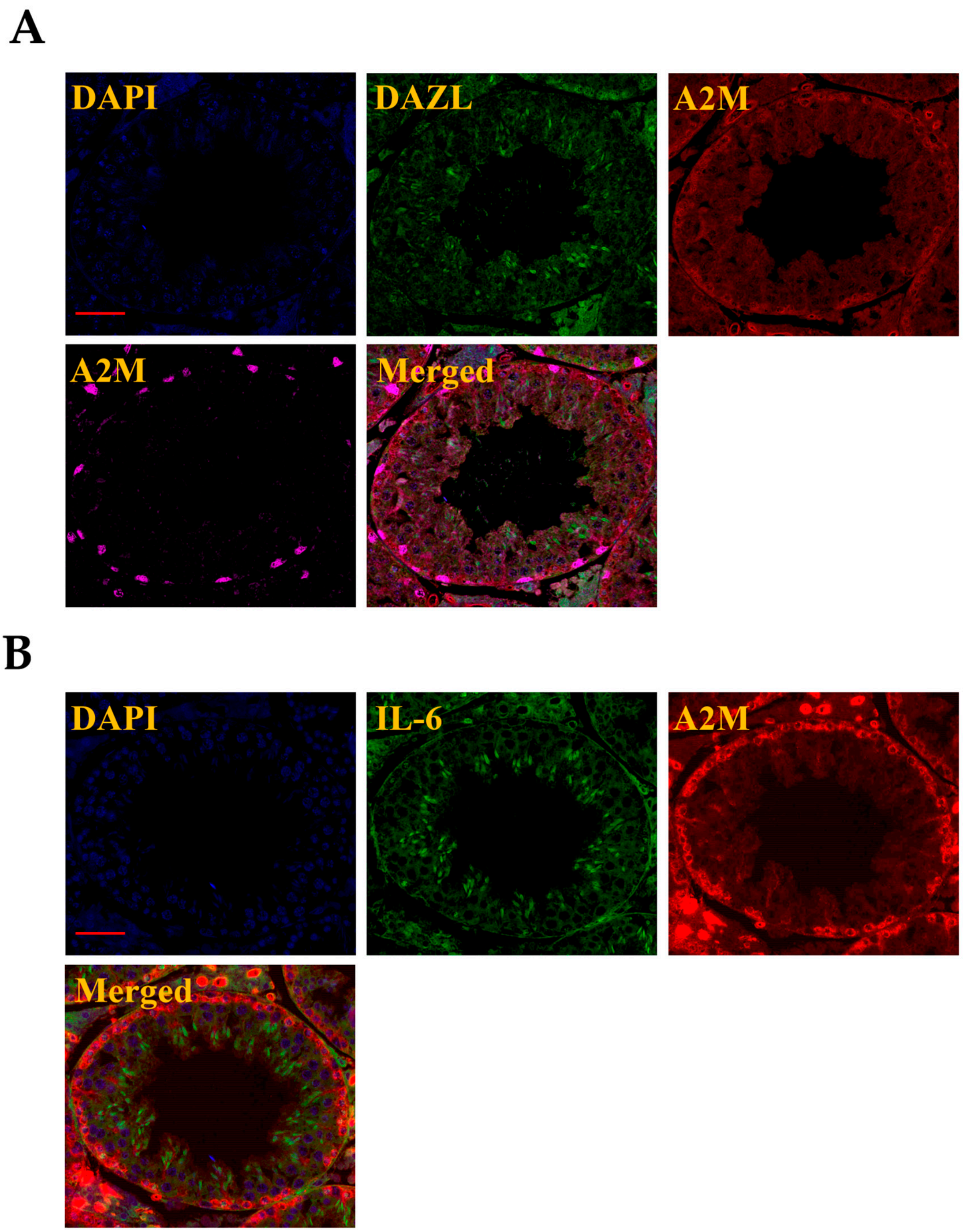

Figure S3. Triple immunofluorescence staining for Sertoli cells (SOX9), spermatocytes (DAZL), and A2M (A), and double immunofluorescence staining with for IL-6 and A2M in the testes of mice from the control group at PND56 (B). Scale bar: 50 μm.

TF	Pattern name	Source	Sequence name	Start	Stop	Strand	Score	P value	Q value	Matched motif
ESR1	m-dataset-1117-2	hTFtarget	mm39_knownGene_ENSMUST00000032203.9	382	397	-	14.3239	2.52e-06	0.00964	AGAGGCAAGGACAGAG
ESR1	m-dataset-1119-2	hTFtarget	mm39_knownGene_ENSMUST00000032203.9	379	403	-	14.7761	3.38e-06	0.00715	AGGAGCAGAGGCAAGGACAGAGGGC
ESR1	m-dataset-1117-4	hTFtarget	mm39_knownGene_ENSMUST00000032203.9	811	821	-	13.9701	3.68e-06	0.0141	AGAGAAGGAGA
ESR1	m-dataset-1119-1	hTFtarget	mm39_knownGene_ENSMUST00000032203.9	393	414	-	7.28169	4.38e-06	0.00875	AACAGACAGGCAGGAGCAGAGG
ESR1	m-dataset-1119-2	hTFtarget	mm39_knownGene_ENSMUST00000032203.9	391	415	-	14.2239	4.83e-06	0.00715	AAACAGACAGGCAGGAGCAGAGGCA
ESR1	m-dataset-1119-2	hTFtarget	mm39_knownGene_ENSMUST00000032203.9	385	409	-	13.9254	5.83e-06	0.00715	ACAGGCAGGAGCAGAGGCAAGGACA
ESR1	m-dataset-1119-1	hTFtarget	mm39_knownGene_ENSMUST00000032203.9	379	400	-	5.74648	7.03e-06	0.00875	AGCAGAGGCAAGGACAGAGGGC
ESR1	m-dataset-1119-2	hTFtarget	mm39_knownGene_ENSMUST00000032203.9	811	835	-	13.4478	7.87e-06	0.00723	AGTGGTGGTTAGACAGAGAAGGAGA
ESR1	m-dataset-1119-1	hTFtarget	mm39_knownGene_ENSMUST00000032203.9	387	408	-	5.15493	8.4e-06	0.00875	CAGGCAGGAGCAGAGGCAAGGA
ESR1	m-dataset-1119-3	hTFtarget	mm39_knownGene_ENSMUST00000032203.9	811	825	+	13.4179	8.78e-06	0.0242	TCTCCTTCTCTGTCT
ESR1	m-dataset-1119-1	hTFtarget	mm39_knownGene_ENSMUST00000032203.9	391	412	-	4.84507	9.21e-06	0.00875	CAGACAGGCAGGAGCAGAGGCA
ESR1	m-dataset-1117-1	hTFtarget	mm39_knownGene_ENSMUST00000032203.9	397	418	-	12.6269	1.15e-05	0.0176	GAGAAACAGACAGGCAGGAGCA
ESR1	m-dataset-1119-2	hTFtarget	mm39_knownGene_ENSMUST00000032203.9	801	825	-	12.7015	1.24e-05	0.00912	AGACAGAGAAGGAGATGAGCAAGGG
ESR1	m-dataset-1119-3	hTFtarget	mm39_knownGene_ENSMUST00000032203.9	383	397	+	12.9552	1.29e-05	0.0242	TCTGTCCTTGCCTCT
ESR1	m-dataset-1117-1	hTFtarget	mm39_knownGene_ENSMUST00000032203.9	393	414	-	12.3881	1.31e-05	0.0176	AACAGACAGGCAGGAGCAGAGG
ESR1	m-dataset-1119-1	hTFtarget	mm39_knownGene_ENSMUST00000032203.9	389	410	-	3.52113	1.35e-05	0.00889	GACAGGCAGGAGCAGAGGCAAG
ESR1	m-dataset-1051-2	hTFtarget	mm39_knownGene_ENSMUST00000032203.9	1722	1735	-	13.3506	1.38e-05	0.0548	GTACAATAAAGTTG

Figure S4. Visualization display of the prediction results of ERE sequences in the mouse *A2m* gene promoter region.

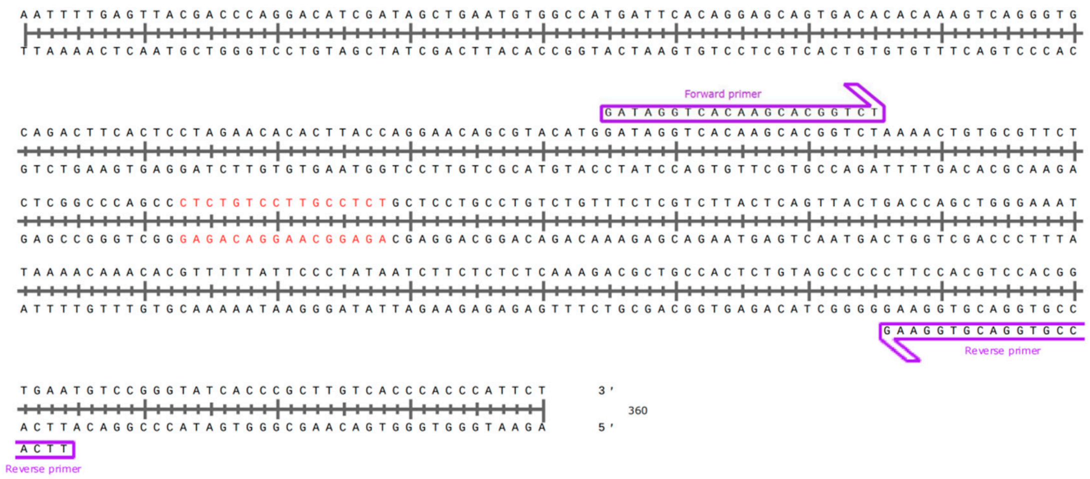

Figure S5. Visualization diagram of the CHIP-qPCR primer sequence design based on the maximum probability ERE theoretical binding site.
